# Zinc and Lead Leaching from Sphalerite–Galena Concentrate Using Deep Eutectic Solvents Based on Choline Chloride: Effect of Roasting and Iodine as Oxidizing Agent

**DOI:** 10.3390/molecules29163742

**Published:** 2024-08-07

**Authors:** Katherine Moreno, Ximena Díaz, Diana Endara, Fernando Sánchez, Carlos F. Aragón-Tobar

**Affiliations:** Department of Extractive Metallurgy, Escuela Politécnica Nacional, Ladrón de Guevara E11-253, P.O. Box 17-01-2759, Quito 170525, Ecuador; xdiaz@me.com (X.D.); diana.endara@epn.edu.ec (D.E.); fernando_fgsr@hotmail.com (F.S.); carlos.aragont@epn.edu.ec (C.F.A.-T.)

**Keywords:** deep eutectic solvents, zinc, lead, leaching, sphalerite–galena concentrate, iodine

## Abstract

The traditional metallurgical routes for producing lead and zinc from primary sources have a significant environmental footprint. Thus, using less pollutant solvents, such as deep eutectic solvents (DESs), would offer a greener solution in metal extraction. This study explores the use of three DESs based on choline chloride (ChCl) (1:2 ChCl–urea, 1:2 ChCl–ethylene glycol, and 1:2 ChCl–glycerol) for recovering Zn and Pb from a sphalerite–galena concentrate of the mining region in Ecuador. Leaching tests of the concentrate (untreated and roasted at 600 °C) in each DES were conducted (30 °C—24 h). The effect of adding iodine as an oxidizing agent was also evaluated. Recoveries of 2% (Zn) and 14% (Pb) were reported when leaching the untreated concentrate with DES. These recovery values increased to 11% (Zn) and 99% (Pb) after adding iodine during the leaching of the untreated concentrate. Roasting had a similar effect on leaching, increasing the recovery values of Zn (75%) and Pb (90%). Combining roasting as a pretreatment and iodine as an oxidizing agent produced higher Zn recoveries (99%) and Pb (99%). These results were compared to recoveries in acid leaching (H_2_SO_4_ and HNO_3_), revealing the potential of DESs as an alternative for metal recovery from primary sources.

## 1. Introduction

Zinc is one of the most commonly used metals after copper and aluminum, and is essential for various applications, notably in the chemical and agricultural industries. The primary ore of zinc is sphalerite (ZnS), which is extensively distributed globally and found in metamorphic, igneous, or sedimentary rocks. More than 95% of zinc production in the world is derived through a process involving roasting, acid leaching, and electrowinning. In this hydrometallurgical recovery treatment, a sphalerite concentrate is calcined, and zinc is leached with sulfuric acid, which is then recovered by electrolysis [1].

Lead, another metal with multiple industrial applications, is primarily used in automobile batteries [2]. Galena (PbS) is the main lead ore, and its extraction is typically performed using pyrometallurgical methods in a blast furnace, employing processes such as Boliden and Outokumpu [3]. However, these processes have environmental drawbacks due to lead dust emissions, which pose significant health risks. There are also hydrometallurgical methods for lead recovery through acid leaching [4].

Both pyrometallurgy and hydrometallurgy have significant environmental footprints and present various challenges [5]. Hydrometallurgy, which uses aqueous acids or alkalis to dissolve metal oxides, sulfides, or silicates [6,7], generates hazardous liquid and solid wastes that require treatment before disposal [5]. Pyrometallurgy faces limitations due to stricter environmental regulations [3].

Extracting metals from primary geological sources and recycling final tailings must adapt to meet the growing demand for metals [8]. A critical challenge for the metallurgical industry is using environmentally friendly, safe, non-toxic, and inexpensive solvents [9].

Solvometallurgy, a technique of extractive metallurgy, offers a new approach for selectively recovering zinc and lead using molecular organic solvents, ionic liquids, or deep eutectic solvents (DESs) instead of aqueous solutions [10]. Solvometallurgy consumes less energy than pyrometallurgy and has shown better selectivity than hydrometallurgy [11].

Among the solvents used in solvometallurgy, DESs are particularly promising. DESs are mixtures of pure compounds, typically formed by a hydrogen bond donor (HBD) and a hydrogen bond acceptor (HBA), where the eutectic point temperature is below that of an ideal liquid mixture [12]. They are advantageous for metal extraction due to their high thermal and chemical stability, lower toxicity, non-flammability, and non-volatility [5].

DESs have been extensively studied over the past 20 years and have numerous applications. They have been used as reaction media in organic and inorganic synthesis, solvents in biomass and biodiesel processing, electrolytes in metal electrodeposition and electropolishing [7], and the preparation of nanostructured metals and alloys [13]. Additionally, they are used in polymer synthesis, metal oxide processing, and the electrodeposition of zinc, tin, and zinc–tin alloys [14,15]. Common bulk chemicals such as urea and oxalic acid are used to form DESs, which have high solubility for a wide range of solutes, including metal oxides [6,7].

Studies have shown that DESs, such as those based on choline chloride and ammonium salt, are non-toxic, biodegradable, and commonly used in household and industrial products [15]. Notable DESs include choline chloride plus urea (reline), choline chloride plus ethylene glycol (ethaline), and choline chloride plus glycerol (glyceline), each mixed in a molar ratio of one mole of choline chloride to two moles of the other component [16]. These solvents are prepared by heating in a sealed container with continuous stirring at 60 °C until a homogeneous and transparent liquid is formed, and then stored at room temperature [5].

Zinc- and lead-rich concentrates in Ecuador are currently exported for refining, with no in-situ recovery options. DESs offer a novel technology for recovering these metals locally due to their low energy consumption and operating costs, utilizing inexpensive inputs such as choline chloride, which is widely used in the agro-industrial sector [17]. Traditional metal recovery methods consume large amounts of energy and generate significant environmental impacts [18], whereas the organic characteristics of DESs provide an ecological solution for mineral processing. Using DESs in metal recovery minimizes environmental impact, significantly reducing the negative effects of effluent or tailings discharge [19].

The application of DESs for the metallurgical recovery of metals from primary sources is a novel topic within small- and large-scale mining in Ecuador, although several studies have indicated that DESs have been used in the recovery of metals from secondary sources with great success, for example in the recycling of lithium-ion batteries for the recovery of lithium and cobalt [20], residual lead from lead battery slags [21], and zinc from steel mill dust. They have also effectively removed heavy metals such as lead and other elements such as sodium, potassium, magnesium, and calcium from various environmental matrices [22,23]. In addition, DESs have been applied to extract precious metals such as gold, silver, and palladium [24]. This provides an excellent scenario for experimenting with mineral concentrates.

Research shows that various metal oxides (MnO_2_, MnO, Fe_2_O_3_, Fe_3_O_4_, Co_3_O_4_, CoO, NiO, CuO, Cu_2_O, ZnO, and PbO) are electrochemically active in DESs, allowing the recovery of metals from these oxides in pure form [5]. Studies also indicate the dissolution of rare earth carbonates (Y, La, Ce, Nd, and Sm) in DESs prepared with choline chloride and urea, malonic acid, and citric acid [25]. Specific studies on zinc, lead, and iron from sulfides have demonstrated the recovery of these metals using mixtures of choline chloride with ethylene glycol and lactic acid, although the results showed higher selectivity for iron compared to zinc and lead [26].

Another study demonstrated the dissolution of various metals (Au, Ag, and Pd) from mineral samples using iodine as an oxidizing agent within DES (ethaline) [27]. Iodine is a highly effective oxidizing agent that has been utilized for the digestion of metals and minerals. It possesses sufficient strength to dissolve gold in aqueous solutions [28,29] and DES systems [27,30,31], under mild conditions, along with other metals found in printed circuit boards [32] and minerals [33]. Electrocatalysis has been suggested as a general and efficient technique for metal dissolution and recovery. It has already proven effective in digesting and separating gallium and arsenic from semiconductors using iodine in a choline chloride: ethylene glycol solution [31].

With this background, the current study will employ optimal conditions to evaluate the leaching of zinc and lead from sphalerite and galena concentrate from the Ecuadorian mining area of Chinapintza, using three different DESs (reline, ethaline, and glyceline).

## 2. Results

### 2.1. Physical, Chemical, and Mineralogical Characterization of the Concentrate

The concentrate from the Chinapintza mining area is the tailing from a previous cyanidation process for recovering gold and silver.

Table 1 presents the chemical characterization of the material used in this research, resulting from X-ray fluorescence (XRF) analysis.

Table 2 shows the mineralogical characterization of the concentrate through X-ray diffraction (XRD) analysis.

Table 1 and Table 2 show the chemical and mineralogical characterization results, respectively. The analyzed sample has a zinc content of 20% and sulfur content of 12%, indicating the presence of zinc ore, primarily sphalerite (63%), and other associated sulfides of iron (pyrite 8%) and lead (galena 9%), as observed in Table 2. The presence of silicates and aluminosilicates, which would form a gangue composed of quartz (6%) and plagioclases (5%), is also noted.

The XRD analysis confirms the predominant presence of sulfides (~80%), such as sphalerite, galena, and pyrite. However, the analysis does not provide information on the amount of iron or other trace elements present in sphalerite. It is crucial to highlight that those mineral deposits rich in sphalerite, such as those in Chinapintza, contain metals such as Fe, Pb, Ga, and Ge associated with this mineral. It is worth noting that the calcium carbonate (CaCO_3_) mentioned in Table 2 could correspond to rhodochrosite (MnCO_3_), as manganese was detected in the XRF analysis.

### 2.2. Preliminary Leaching Tests with DESs

**Figure 1:**
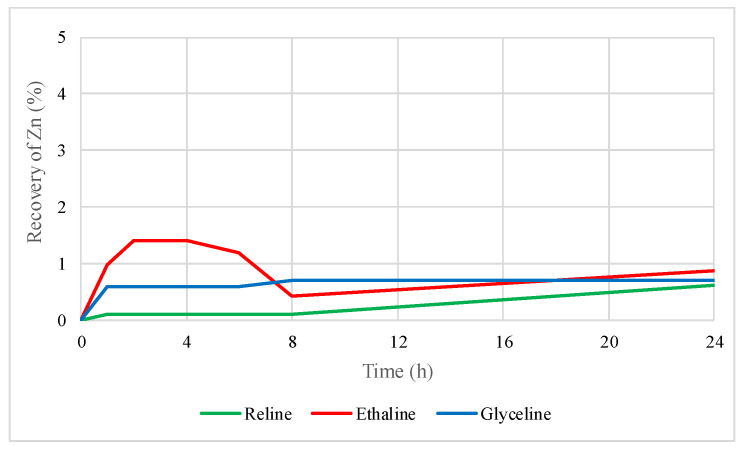
Zinc recovery by leaching sphalerite–galena concentrate using reline, ethaline, and glyceline.

Figure 1 shows the zinc recovery from the concentrate using the three DESs; the recovery percentage of zinc does not exceed 2% after 24 h. The recovery was 0.6% for reline, 1% for ethaline, and 0.7% for glyceline. These results indicate that neither of the three DESs efficiently recovered zinc from the sulfide form. According to the results presented by [26], the low zinc recovery observed is caused by a greater affinity for iron within the sphalerite. During the first eight hours, ethaline solubilized a higher amount of zinc compared to reline and glyceline.

**Figure 2:**
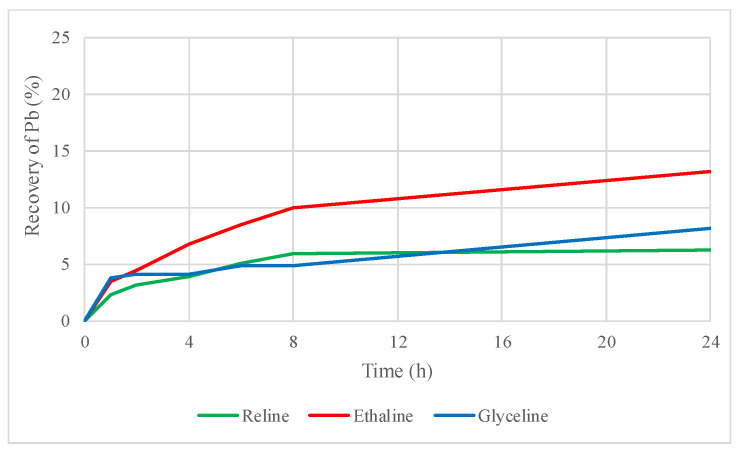
Lead recovery by leaching sphalerite–galena concentrate using reline, ethaline, and glyceline.

As shown in Figure 2, lead has a higher affinity in the three tested DESs, reaching 6% recovery with reline, 13% with ethaline, and 8% with glyceline.

The preliminary leaching test with DESs led to low recoveries in both metals (zinc with 1% and lead with 13%). An oxidizing agent within DES could be an alternative to increase metal recovery. Therefore, the following section includes iodine as an oxidizing agent for better zinc and lead recoveries during DESs leaching.

### 2.3. Influence of Oxidizing Agent in the DES Leaching Process

The study of [27] demonstrated that various metals can be dissolved from ore samples using iodine (I_2_) as the oxidizing agent within a DES. The gold ore they used has a gangue composed mainly of pyrite (Fe_2_S) and some base metal sulfites. The use of iodine in DES leaching showed a strong selectivity of DES in the fast dissolution of electrum, so this technique has a high potential to separate gold from pyrite. Based on this study, an attempt will be made to use iodine within the composition of a DES to increase the dissolution of other metals.

**Figure 3:**
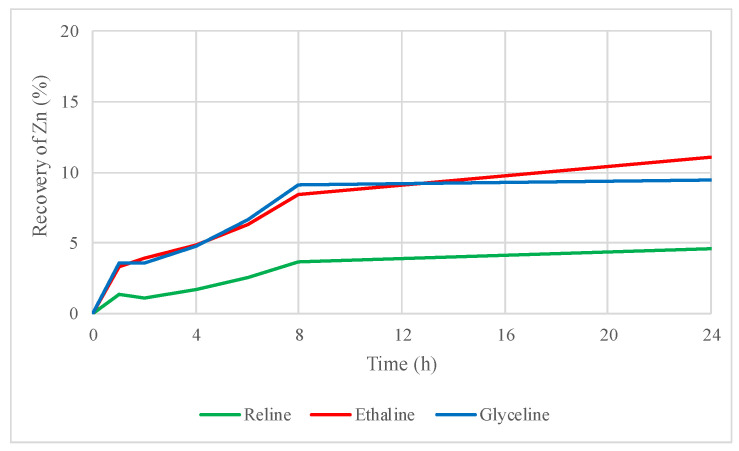
Zinc recovery by leaching sphalerite–galena concentrate using reline, ethaline, glyceline, and iodine as an oxidizing agent within DES.

**Figure 4:**
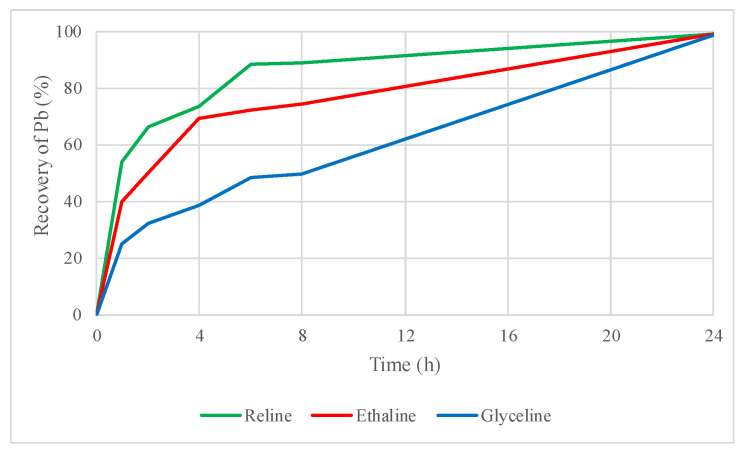
Lead recovery by leaching sphalerite–galena concentrate using reline, ethaline, glyceline, and iodine as an oxidizing agent within DES.

Figure 3 and Figure 4 show that zinc and lead recoveries from sphalerite and galena concentrate improved by adding the oxidizing agent iodine dissolved in the DES, respectively.

In the case of zinc, recoveries increased from 0.6% to 5% for reline, from 1% to 11% for ethaline, and from 0.7% to 9% for glyceline, demonstrating a better dissolution of the concentrate when iodine is added as oxidizing agent in leaching tests with DESs. The improvement in lead recovery was even more noticeable when iodine was used as an oxidizing agent. In fact, lead solubility in these DESs reaches almost 100% in the three DESs within the first 24 h. Lead recoveries increased from 6% to 99% with reline, 13% to 99% with ethaline, and 8% to 99% with glyceline. The addition of iodine in the leaching test was beneficial overall; for instance, more than 50% of dissolved lead was recovered from the sample only in the first hour of leaching with reline plus iodine.

Metal solubility in DES depends on the mineral form in which the metal is found [17]. Changes in mineralogy produced by thermal treatment could also improve metal recovery in the same way as adding an oxidizing agent. Therefore, in the next section, the effect of thermal treatment on metal recovery is explored.

### 2.4. Influence of Thermal Treatment of the Concentrate in the DES Leaching Process

#### 2.4.1. Establishing the Best Roasting Conditions for the Concentrate

Roasting is one of the previous steps in the metal recovery process [34]. Several studies, such as those of [5,35], indicate that metals show better solubility in DES when they are in the form of oxide or sulfate, compared to the limited solubility of sulfides in DESs. Because sulfides are found in the concentrate, roasting could be an alternative for increasing metal recovery in leaching tests with DES.

One of the challenges of this research is achieving the temperature and residence time for a roasting process that ensures the oxidation of the sulfides in the concentrate. In the case of sphalerite, it is desired that zinc does not volatilize, as zinc is a metal that tends to volatilize as temperature increases. Moreover, if the roasting process is not controlled, there is a risk of converting ZnS into zinc ferrite (ZnFe_2_O_4_), which does not leach in a weakly acidic solution. In the case of galena, since it is a more stable compound, it would be treated with the temperature and time proposed for sphalerite.

Previous research [36] indicates that in various forms of ZnS, including natural forms such as sphalerite and highly crystalline materials, oxidation experiments have been conducted over a wide range of temperatures from 500 to 1440 °C, and the point at which diffusion becomes significant varies between 600 and 830 °C. The most interesting results of this study were obtained by using low volumes of oxygen at temperatures between 480 and 600 °C, with good recoveries.

**Figure 5:**
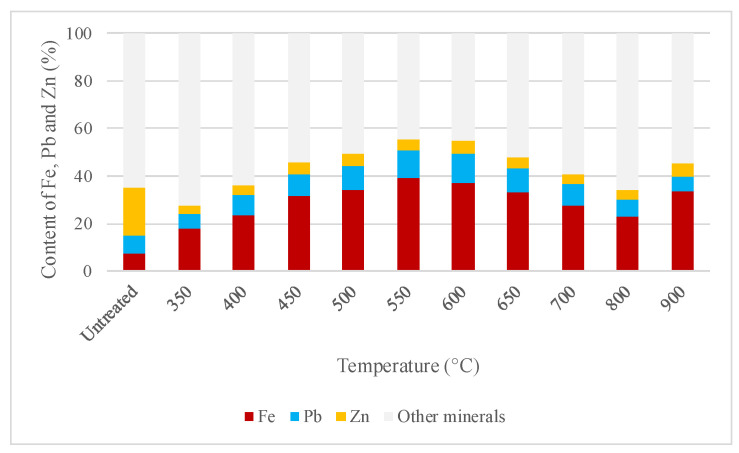
Iron, lead, and zinc content (%) in the sphalerite and galena concentrate, in the head sample, and all samples at the employed temperatures.

Based on the conditions presented above, Figure 5 shows the results of the Fe, Pb, and Zn content by XRF of the samples roasted at different temperatures between 350 °C and 900 °C for 8 h to oxidize all the sulfides contained in the studied concentrate and to determine the best conditions for the applied thermal treatment.

Based on the data presented in Figure 5, it is determined that the optimal roasting temperature is 600 °C, a finding corroborated by [36]. This temperature was chosen because it yielded the highest zinc and lead contents obtained (5% Zn and 12% Pb) compared to other temperatures. The sphalerite content decreased significantly from 63% to 6%, and pyrite from 8% to 6%, while hematite content increased in the roasted sample to 66%. Based on this composition, iron found in the hematite would come mainly from the iron oxidation in sphalerite and, to a lesser extent, from the iron oxidation of pyrite. This phenomenon can be explained by the stability of pyrite as an iron sulfide compared to other iron-containing sulfides, such as sphalerite, pyrrhotite, and chalcopyrite, which readily react to form other compounds [37]. Sphalerite is still present in the roasted product, and no other zinc species were found. The limited solubility of zinc from sphalerite compared to other more soluble zinc species needs to be considered in the following analysis of the leaching tests. Importantly, galena could oxidize to anglesite, resulting in significant lead recoveries.

#### 2.4.2. Leaching Tests of the Roasted Concentrate with DESs

**Figure 6:**
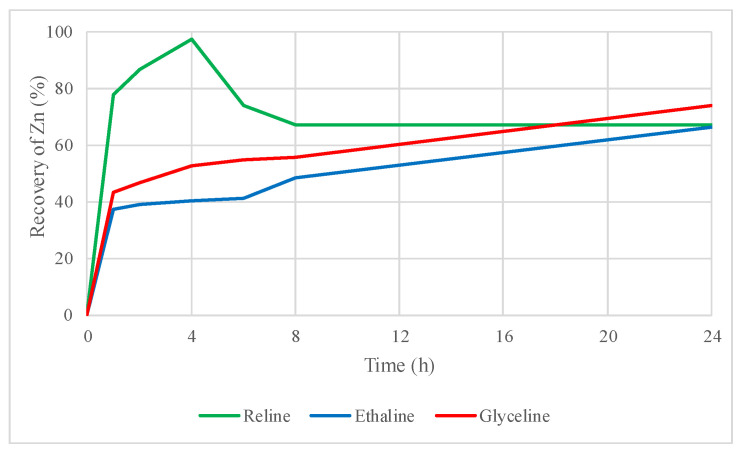
Zinc recovery by leaching of the roasted concentrate using reline, ethaline, and glyceline.

**Figure 7:**
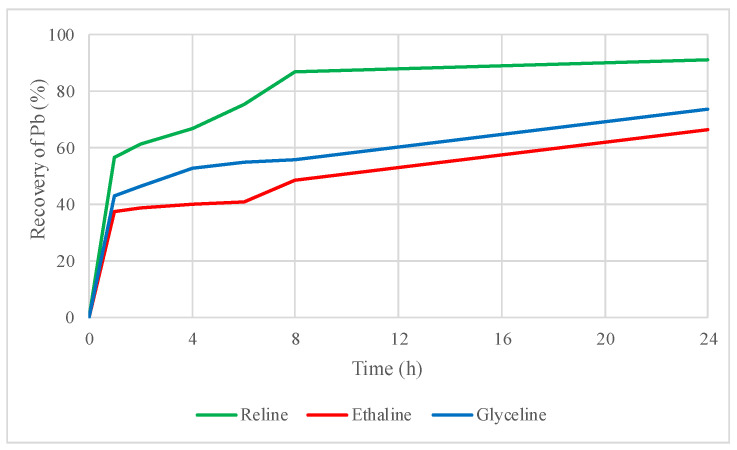
Lead recovery by leaching of the roasted concentrate using reline, ethaline, and glyceline.

Figure 6 and Figure 7 present the recovery of zinc and lead from the leaching of the roasted concentrate with three DESs.

Zinc recoveries increased from 0.6% to 67% with reline, 1% to 66% with ethaline, and 0.7% to 74% with glyceline. These improvements in the zinc recoveries are directly related to the previous thermal processing (roasting at 600 °C). However, it is important to note that the sample is no longer a concentrate rich in sphalerite and galena due to roasting, but instead a hematite concentrate (66% Fe_2_O_3_). The content of sphalerite after the thermal treatment decreased from 63 to 6%. This variation in the sphalerite content has been related to iron oxidation in the mineral, leaving mainly the zinc associated with the sulfide in the structure. It is possible that the remaining zinc in the sulfide structure is more prone to dissolution once iron has been removed thanks to the thermal process, thereby explaining the higher recoveries presented in Figure 6. 

Lead recoveries increased from 6% to 91% with reline, 13% to 47% with ethaline, and 8% to 62% with glyceline. Leaching with reline reached the higher lead recoveries once a thermal treatment was applied to the concentrate. It is important to mention that ethaline reached a higher lead recovery when the untreated concentrate was leached. However, in the leaching of the roasted concentrate, leaching with ethaline only increased the lead recovery to 47% compared to the 91% obtained with reline. 

#### 2.4.3. Leaching Tests of the Roasted Concentrate with DESs plus I_2_ as an Oxidizing Agent

**Figure 8:**
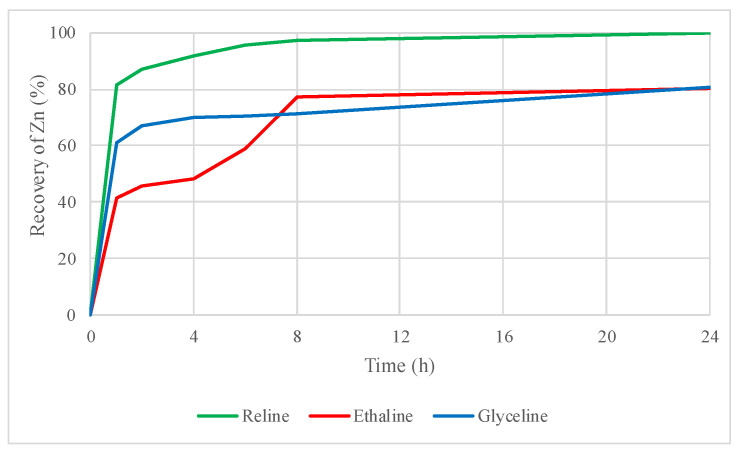
Zinc recovery by leaching the roasted concentrate using reline, ethaline, and glyceline, with iodine as an oxidizing agent.

In Figure 8, the recovery values of zinc from the roasted concentrate in DES leaching tests with I_2_ as an oxidizing agent are presented. Thanks to the addition of iodine as an oxidizing agent, higher zinc recovery values were obtained, for instance, in the case of reline, recoveries increased from 67% to 99%. Similar increases in the recovery values were observed in ethaline (from 66% to 80%) and glyceline (74% to 80%).

**Figure 9:**
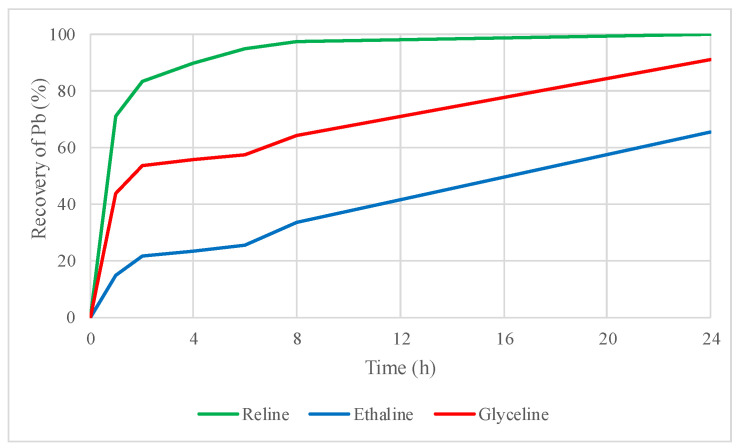
Lead recovery by leaching the roasted concentrate using reline, ethaline, and glyceline, with iodine as an oxidizing agent.

Figure 9 shows the lead recovery from the leaching tests of the roasted concentrate with DES, using iodine as an oxidizing agent. Thanks to the addition of iodine, the recovery results for this metal with reline increased from 91% to 100%. With ethaline, the lead recovery from the roasted concentrate increased from 47% to 66%. With glyceline, the recovery increased from 62% to 91%.

### 2.5. Evaluation of Zinc and Lead Recovery by DES Leaching with the Influences of Thermal Treatment and the Addition of Iodine as an Oxidizing Agent

**Figure 10:**
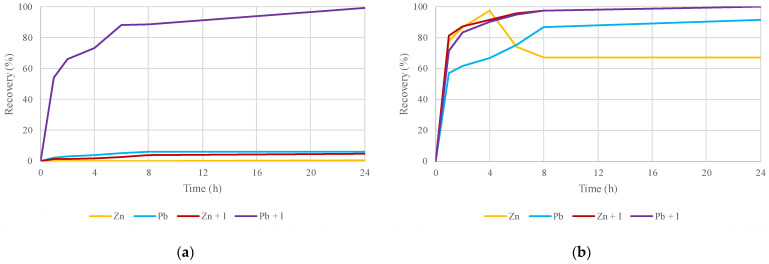
Zinc and lead recoveries by leaching a sphalerite–galena concentrate using reline with and without adding oxidizing agent I_2_: (**a**) untreated concentrate; (**b**) roasted concentrate.

Figure 10 evaluates the effect of the thermal treatment and the oxidizing agent on the metal recovery from the concentrate using reline as a leaching agent. Reline is taken as an example because of the higher recoveries obtained with this DES compared to ethaline and glyceline. On the left side, Figure 10a compares the zinc and lead recoveries from the sphalerite–galena concentrate using reline as the leaching agent, with and without adding iodine as an oxidizing agent. On the right side, Figure 10b compares the zinc and lead recoveries using reline in a sample of the roasted concentrate, with and without adding iodine as an oxidizing agent.

For the sphalerite–galena concentrate, adding iodine in the leaching test with reline increased zinc recovery from 0.62% to 5%, and for lead, it increased from 6% to 99%. Adding iodine was only beneficial for improving the lead recovery, with less impact on the zinc recovery. For the roasted concentrate, adding iodine in the leaching test with reline increased the zinc recovery from 67% to 99%, and the lead recovery increased from 91% to 99%. For both metals, the leaching of the roasted concentrate with reline improved with the addition of iodine as an oxidizing agent, with recoveries of 99% for zinc and lead.

Table 3 and Table 4 summarize the best zinc and lead recoveries obtained from leaching tests at different experimental conditions. These results are compared with the metal recovery obtained in conventional acid leaching (sulfuric acid for zinc and nitric acid for lead).

The best leaching conditions with DESs were with reline as a leaching agent and adding iodine using the roasted concentrate, as recoveries of 99% zinc and 100% lead were obtained. These recovery values are higher than metal recovery using traditional acid leaching (96% of zinc recovery in H_2_SO_4_ and 80% of lead recovery in HNO_3_).

## 3. Discussion

### 3.1. Influence of Mineralogy in the DES Leaching Process

The results obtained in the preliminary leaching tests with DES of the sphalerite–galena concentrate were analyzed. It is highlighted that lead is found in the form of anglesite (PbSO_4_ with 13%) and galena (PbS with 9%), with lead sulfate (anglesite) being a more soluble species compared to the lead sulfide (galena). Thus, lead recovery observed in the preliminary test recovery (6–13%) would be related to the dissolution of the most soluble species (anglesite), instead of lead in the form of galena. In the case of zinc, the recoveries reach up to 1% with all the DESs, evidencing a limited dissolution of zinc. Considering that zinc is only found in the form of sulfide, the low recoveries of this metal in the preliminary leaching tests respond to the limited solubility of sphalerite in the DESs used, as has been observed in [17]. Comparing the three DESs used in the leaching tests, ethaline shows higher metal recoveries (1% Zn and 13% Pb) than reline and glyceline. Moreover, lead showed higher affinity than zinc during the leaching tests in the three DESs used. 

The recovery of zinc and lead from a sulfide concentrate using DESs largely depends on the mineral species in which these metals are found. Therefore, the mineralogy of the concentrate directly influences the selectivity of DESs in metal recovery.

The dissolution mechanism of zinc and lead from sulfides in the three DESs analyzed requires a deeper understanding of the speciation of these two metals during leaching. Although the scope of this study does not contemplate a fundamental analysis of these interactions between metals and DES, some valuable information regarding this behavior can be obtained from the literature. For instance, in the study of [38], electrochemical measurements were employed to determine the mechanism of zinc dissolution from sphalerite concentrate in a ternary deep eutectic solvent (choline chloride, p-toluene sulfonic acid, and ethylene glycol). This experimental approach of [38] was complemented with molecular dynamic (MD) simulation to represent the interaction between zinc and iron ions with the anionic species of the three-component DES. Therefore, the leaching of sphalerite in DES resulted in the formation of zinc chloride ion complexes and elemental sulfur/sulfate ions. In fact, metal cations tend to form complexes predominantly with the chlorine atom of choline chloride in DES leaching, as it has also been corroborated in dissolution studies of other sulfides such as chalcopyrite [39]. Based on these findings, it can be stated that the presence of different zinc chloride complexes, such as [ZnCl_3_^−^]^−^ or [ZnCl_5_^−^]^3−^, would characterize the leaching process of the concentrate presented in this study. A similar situation could be observed for lead with the formation of metallic chloride complexes. The formation of these chloride complexes appears to be more favorable than other complexes during the dissolution process of sphalerite in DES.

### 3.2. Influence of the Oxidizing Agent in the DES Leaching Process

The use of iodine as an oxidizing agent during the leaching test with the three DESs showed encouraging results in the recovery of zinc and lead from the concentrate. In the study of [40], the role of iodine as a catalyst for the dissolution and recovery of metals in DESs is presented. These authors indicate that iodine is not forming chemical bonds with any of the DES components in detectable quantities and that iodinated organic compounds are not being generated. This catalytic effect of iodine within the DES–metal reaction is responsible for the increase observed in the dissolution of both metals in the three DESs used (recoveries of 5% Zn and 99% Pb in reline, 11% Zn and 99% Pb in ethaline, and 9% Zn and 99% Pb in glyceline). In fact, as it was previously pointed out by [31], the reductive potential of iodine compared to the oxidative potential of zinc and lead is higher, which should allow the oxidative leaching of these elements. The lead concentration in the solution increases more rapidly than that of zinc, which is consistent with the greater thermodynamic driving force for iodine to oxidize lead. As mentioned by [31], this would be the redox reactions of iodine into DES (1):

I_2_ + 2 e^−^ = 2 I^−^ (1)

I_2_ + I^−^ = I_3_^−^ (2)

I_3_^−^ + 2 e^−^ = 3 I^−^ (3)

### 3.3. Influence of Thermal Treatment in the DES Leaching Process

Applying thermal roasting treatment to the mineral concentrate improves zinc recoveries. However, it is important to note that the sample ceases to be a concentrate rich in sphalerite and galena, transforming into a hematite (Fe_2_O_3_) concentrate during roasting. This results in a significant decrease in zinc concentrations, making it extremely soluble in the DESs, which is reflected in the high percentages of zinc recovery (Figure 6). In contrast, lead increases its concentration in the form of sulfate, making it preferentially more soluble than zinc in the DESs used in this study.

It is essential to note that the roasted concentrate does not show the presence of zinc sulfates or oxides. This metal did not oxidize completely with the thermal treatment provided, but the sphalerite present in the concentrate probably underwent slight purification when the iron impurity oxidized. To produce the oxidation of zinc in the concentrate, we suggest applying a more efficient thermal treatment such as oxidative roasting in a fluidized bed furnace to produce zinc oxide, which would improve the recoveries of this metal from the roasted concentrate using leaching with DESs. 

### 3.4. Influence of the Oxidizing Agent and Thermal Treatment at the Same Time in the DES Leaching Process

When the oxidizing agent iodine is applied in the DES leaching of a roasted concentrate, recoveries improve significantly compared to the leaching test with the untreated concentrate. Zinc is recovered almost entirely after 24 h of leaching, as is lead with reline. Similarly, ethaline and glyceline achieve excellent recovery percentages for both metals, making this methodology an interesting alternative for the recovery of zinc and lead.

Inadequate roasting conditions may result in incomplete oxidation, leaving residual sulfides that are more difficult to dissolve, thereby reducing recovery efficiency. Conversely, optimal roasting conditions create a more reactive concentrate, which responds better to leaching agents such as reline, especially when enhanced with iodine. This is evident in the excellent improvement in metal dissolution from the roasted concentrate, where zinc and lead recoveries reached almost 100%.

### 3.5. Comparison with Conventional Acid Leaching Routes

The concentration of sulfuric acid plays a crucial role in the recovery of metals in conventional hydrometallurgical processes. Compounds such as sulfides tend to be poorly soluble in many mediums, and as previously mentioned, if the concentrate has not been roasted under specific conditions, subsequent processes will be affected. At low concentrations of sulfuric acid (less than 1 M), the dissolution of zinc is practically insignificant, and impurities in sphalerite, such as iron, passively precipitate on the surface of pure sphalerite particles, hindering their dissolution.

On the other hand, nitric acid proves effective in dissolving lead as long as an appropriate concentration is maintained (1–2 M). However, when the concentration exceeds 2 M, the acid itself oxidizes sulfides to elemental sulfur or sulfate due to its strong oxidizing nature. This leads to increased reagent consumption, leaching treatment, and acid regeneration costs.

The optimal zinc recovery method occurred through the use of the eutectic solvent ethaline, particularly when this metal is in sulfide form. If not, the preferred solvent is reline. It is observed that ethaline holds a slight advantage over sulfuric acid as it exhibits better zinc recovery; ethaline dissolves zinc by nearly 12%, whereas sulfuric acid reaches 10%.

In lead recovery, the results are similar to those of zinc. Almost 100% recovery is achieved in the sample without any prior thermal treatment when using reline and the oxidizing agent iodine, suggesting a high selectivity of this DES towards lead, dissolving it virtually completely. In fact, all three DESs show high selectivity for lead, with excellent recoveries (reline 100%, ethaline 99%, glyceline 99%); in comparison, leaching in the acidic medium of this sample under the same conditions only achieves a 20% recovery. Therefore, lead solvometallurgy could be considered a cost-effective and environmentally suitable alternative. High lead recovery values are obtained for the roasted sample in both leaching tests. Lead is found in the form of sulfate (anglesite) due to galena (PbS) alteration, which apparently favors the recoveries, which tend to be higher. 

## 4. Materials and Methods

### 4.1. Characterization of the Concentrate

The concentrate samples were taken directly from the precious metal recovery plant in the Zamora Chinchipe province in southeast Ecuador.

The samples were dried, homogenized, and quartered into 1 kg fractions for further testing.

X-ray fluorescence analysis was performed using the Bruker S8 Tiger instrument (Bruker, Karlsruhe, Germany) to determine the chemical composition of the concentrate. X-ray diffraction was performed using the Bruker AXS D8 Advance model (Bruker, Karlsruhe, Germany) to determine the mineralogical composition.

The elemental composition of Cu, Zn, Pb, Fe, Au, Ag, As, Si, Ca, Al, among others, was determined using atomic absorption spectrophotometry on a Perkin Elmer AA 300 instrument (Perkin Elmer, Shelton, CT, USA) and inductively coupled plasma mass spectrometry (ICP-MS) on an Agilent 7500e ICP-MS instrument at the University of Utah (Agilent, Santa Clara, CA, USA). Prior to analysis, the concentrate was digested using hydrochloric and nitric acids in a Milestone ETHOS ONE microwave oven (Milestone Srl, Milan, Italy).

### 4.2. Thermal Treatments of Sulfide-Rich Concentrate

Considering that the recovery of zinc and lead is improved when these elements are in the form of oxides in the mineral [37], the sample was roasted at various temperatures and times to determine the best thermal conditions for pretreatment. Oxidative roasting tests were conducted at various temperatures from 350 to 900 °C for 2, 4, 6, and 8 h. The thermal treatment was used to convert the zinc and lead sulfides from the original sample into oxides and sulfates in order to evaluate whether the recoveries of the metals of interest in this study improve with a roasted sample. The best thermal condition, i.e., the roasting temperature and time where the calcine presents the highest zinc and lead content in the analyzed sample, was chosen, given that these elements, especially zinc, can volatilize with thermal treatment.

The zinc, lead, and other element contents were analyzed after the thermal treatment using X-ray fluorescence chemical analysis with the Bruker S8 Tiger instrument (Bruker, Karlsruhe, Germany) for each roasting temperature and time.

### 4.3. Leaching Tests Using DESs

Ref. [9] suggest the preparation of three DESs, in which analytical grade choline chloride at 98% purity from Sigma Aldrich serves as the hydrogen bond acceptor (HBA), combined with hydrogen bond donor (HBD) structures such as analytical grade urea at 99% purity from Sigma Aldrich, analytical grade ethylene glycol at 99% purity from Mallinckrodt, and analytical grade glycerin at 99.5% purity from Fischer Scientific. Thus, reline was prepared, which is the eutectic solvent formed by choline chloride and urea, with 1 mole of choline chloride and 2 moles of urea, meaning a molar ratio of 1:2. This combination was mixed by agitation and heating to 60 °C until the two solid reactants formed a transparent liquid [17]. Similarly, the other eutectic solvents, ethaline consisting of choline chloride and ethylene glycol, and glyceline consisting of choline chloride and glycerin, were prepared in the same molar ratio of 1:2 of choline chloride with ethylene glycol or glycerol, respectively [13].

Subsequently, other experiments were conducted using iodine (I_2_) as an oxidizing agent within the reaction, which was employed immediately before the experiments in a ratio of 100:1 of the DES with respect to I_2_, following the procedure proposed by [27,31].

The leaching tests with the DESs (reline, ethaline, and glyceline) were performed on sphalerite and galena concentrate samples, with and without thermal treatment.

As suggested by [41], for each leaching test with the DESs, 0.2 g of concentrate was used in 10 g of each DES, in this case, reline, ethaline, and glyceline, respectively, with the addition of the corresponding mass of I_2_.

Following the methodology suggested by [42] to determine the zinc and lead concentrations in the DESs, 100 µL of solution samples were taken at times 1, 2, 4, 6, 8, and 24 h. These samples were diluted in a 50 mL volumetric flask using a 2% HNO_3_ solution for subsequent analysis by atomic absorption to quantify the metals dissolved in the DES, performing atomic absorption analysis with a Perkin Elmer AA 300 instrument (Perkin Elmer, Shelton, CT, USA).

### 4.4. Leaching Tests Using Acids

Two types of acid solutions were prepared with a concentration of 150 g/L for each solution. Analytical grade sulfuric acid with 98% purity from the Fisher brand was used for zinc leaching tests, while 70% purity nitric acid from the Fisher brand was employed for lead leaching tests.

Leaching tests were conducted with sphalerite and galena concentrate samples, with and without thermal treatment, using 100 g samples of 10% solids for 24 h with mechanical agitation.

The concentrations of zinc and lead in the enriched solution and wash solution were determined by atomic absorption using a Perkin Elmer AA 300 instrument (Perkin Elmer, Shelton, CT, USA).

The leaching residue cakes were dried and homogenized, and then 0.2 g portions were taken for acid digestion (with nitric, hydrofluoric, and hydrochloric acid). Once the acid solutions with the digested residue were obtained, zinc and lead elements were measured by atomic absorption using a Perkin Elmer AA 300 instrument (Perkin Elmer, Shelton, CT, USA).

## 5. Conclusions

Mineralogical analysis of the concentrate confirmed the presence of sulfides, such as sphalerite (63%), a zinc-bearing mineral; galena (9%) and anglesite (7%), lead-bearing minerals; and pyrite (8%), an iron-bearing mineral. Chemical analysis corroborated the presence of the components of these sulfides: Zn (20%), Pb (7%), Fe (7%), and S (12%). 

Preliminary leaching tests of the sphalerite–galena concentrate in DESs (reline, ethaline, and glyceline) were unsatisfactory due to the low recoveries of zinc (<1%) and lead (<13%). Recovery of zinc in DESs was low due to the limited solubility of sphalerite, the only zinc-bearing species. Lead recovery was higher because of the presence of anglesite, a lead sulfate more prone to dissolve in DES, compared to galena, the other lead-bearing mineral found in the concentrate. These findings indicate a strong relationship between the mineralogy and the selectivity of DES.

The use of iodine as an oxidizing agent during leaching tests with DESs demonstrated a significant improvement in the recovery of zinc and lead. Higher metal recoveries were reported (5% Zn and 99% Pb in reline + I_2_, 11% Zn and 99% Pb in ethaline + I_2_, and 9% Zn and 99% Pb in glyceline + I_2_). These findings confirmed the high oxidative potential of iodine, effectively increasing the recovery values of both metals.

Thermal roasting treatment (at an optimal temperature of 600 °C) improved the recovery of zinc and lead in the three DESs studied. Zinc recovery increased to 67% in reline, 66% in ethaline, and 74% in glyceline. Lead values increased to 91% in reline, to 47% in ethaline, and to 62% in glyceline. Thus, thermal treatment produced a beneficial effect due to the changes in more soluble species of lead and zinc induced by roasting.

Combining the thermal treatment of the concentrate with the oxidizing agent (I_2_) in the leaching tests with DESs produced the best recovery values in both metals. For instance, a 99% recovery of zinc and a 100% recovery of lead were obtained by leaching the roasted concentrate with reline + I_2_. 

Compared to conventional acid leaching, the leaching of the roasted concentrate with DES + I_2_ showed better metal recoveries. In the case of zinc, leaching with reline + I_2_ reached recovery values (99%) comparable to leaching with sulfuric acid (96%). Lead recovery with reline + I_2_ reached higher recovery values (100%) compared to conventional acid leaching with nitric acid (80%). Therefore, the proposed leaching process using reline + I_2_ outperformed traditional acid leaching methods at the laboratory scale. 

## Figures and Tables

**Table 1:** Chemical characterization of the concentrate.

Element	Content (%)
Zn	20
S	12
Pb	8
Fe	7
Si	6
Al	2
Mn	1
K, Ca, Cu, Na and Mg	<1

**Table 2:** Mineralogical characterization of the concentrate.

Mineral	Formula	Content (%)
Sphalerite	ZnS	63
Galena	PbS	9
Pyrite	FeS_2_	8
Anglesite	PbSO_4_	7
Quartz	SiO_2_	6
Muscovite	KAl_2_(AlSi_3_O_10_)(OH)_2_	4
Calcite	CaCO_3_	2
Plagioclase	(Na,Ca)Al(Si,Al)Si_2_O_8_	1

**Table 3:** Zinc recovery (%) with different leaching agents (solid:DES ratio 2%*w*/*w*, 30 °C, and 24 h).

Leaching Agent	Untreated Concentrate	Untreated Concentrate + I_2_	Roasted Concentrate	Roasted Concentrate + I_2_
Reline	0.6	5	67	99
Ethaline	1	11	66	80
Glyceline	0.7	9	74	80
Sulfuric Acid *	10	-	96	-

* Conditions: (150 g L^−1^ of H_2_SO_4_, 10%*w*/*w*, 22 °C, and 24 h).

**Table 4:** Lead recovery (%) with different leaching agents (solid:DES ratio 2%*w*/*w*, 30 °C, and 24 h).

Leaching Agent	Untreated Concentrate	Untreated Concentrate + I_2_	Roasted Concentrate	Roasted Concentrate + I_2_
Reline	6	99	91	100
Ethaline	13	99	47	66
Glyceline	8	99	62	91
Nitric Acid *	22	-	80	-

* Conditions: (150 g L^−1^ of HNO_3_, 10%*w*/*w*, 22 °C, and 24 h).

## Data Availability

Data are contained within the article.
